# Literature Cases Summarized Based on Their Polysomnographic Findings in Rett Syndrome

**DOI:** 10.3390/ijerph19063422

**Published:** 2022-03-14

**Authors:** Xin-Yan Zhang, Karen Spruyt

**Affiliations:** Université de Paris, NeuroDiderot INSERM, 75019 Paris, France

**Keywords:** Rett syndrome, sleep problems, sleep study, total sleep time, rapid eye movement sleep

## Abstract

Rett syndrome (RTT) is a severe and rare neurodevelopmental disorder affecting mostly girls. In RTT, an impaired sleep pattern is a supportive criterion for the diagnosis, yet little is known regarding the sleep structure and sleep respiratory events. Aiming to delineate sleep by aggregating RTT case (series) data from published polysomnographic studies, seventy-four RTT cases were collected from eleven studies up until 6 February 2022 (PROSPERO: CRD 42020198099). We compared the polysomnographic data within RTT stratifications and to a typically developing population. *MECP2* cases demonstrated shortened total sleep time (TST) with increased stage N3 and decreased REM sleep. In cases with *CDKL5* mutations, TST was longer and they spent more time in stage N1 but less in stage N3 than those cases affected by *MECP2* mutations and a typically developing population. Sleep-disordered breathing was confirmed by the abnormal apnea/hypopnea index of 11.92 ± 23.67/h TST in these aggregated cases. No association of sleep structure with chronological age was found. In RTT, the sleep macrostructure of *MECP2* versus *CDKL5* cases showed differences, particularly regarding sleep stage N3. A severe REM sleep propensity reduction was found. Aberrant sleep cycling, possibly characterized by a poor REM ‘on switch’ and preponderance in slow and high-voltage sleep, is proposed.

## 1. Introduction

Rett syndrome (RTT, OMIM # 312750) is a rare neurodevelopmental disorder predominantly occurring in females [1]. Mutations in genes such as methyl-CpG-binding protein-2 (*MECP2*) [2], cyclin-dependent kinase-like 5 (*CDKL5*) [3] and forkhead box protein G1 (*FOXG1*) [4] are considered pathogenic, of which *MECP2* is responsible for more than 90% of the cases [5]. Abnormal characteristics of RTT usually become visible after months of “normal” development followed by profound regression, such as loss of functional hand skills, spoken language and mobility, as well as the presence of stereotypical hand movements, cognitive disabilities and gait dyspraxia [1,6]. Other comorbidities including breathing disturbances, epileptic seizures and scoliosis are characteristic of RTT. Classic and variant phenotypic RTT are defined by the (partial) presence of the abovementioned clinical features [1]. In addition, clinical manifestations may vary over time. Thus, cases with RTT are also described by the course of their traits, i.e., early-onset stagnation (stage I), rapid developmental regression (stage II), pseudostationary period (Stage III) and late motor deterioration (Stage IV) [7]. 

Disturbed sleep is a supportive diagnostic criteria for RTT [1], with parents and caregivers reporting sleep behaviors such as frequent night awakenings, night laughing, night screaming and difficulties falling asleep [8,9,10]. In the studies applying polysomnography (PSG), a range of sleep structure alterations in RTT have been reported, such as decreased sleep efficiency (SEI), increased number of awakenings, decreased rapid eye movement sleep (REM) and increased non-rapid movement sleep (NREM) stage 3 [11,12,13], but findings are inconsistent [14,15]. In addition, whilst the breathing disturbances during the wake state have been well investigated [16,17], respiratory findings during sleep remain dissimilar in previous studies.

In this study, we aim to perform an analysis of aggregated literature data reporting PSG parameters of RTT cases. This approach may provide more robust findings, allowing generalizations with respect to RTT sleep. We apply a stratified approach per their clinical and mutation characteristics to gain directives for future investigation.

## 2. Materials and Methods

### 2.1. Case Selection

**Figure 1:**
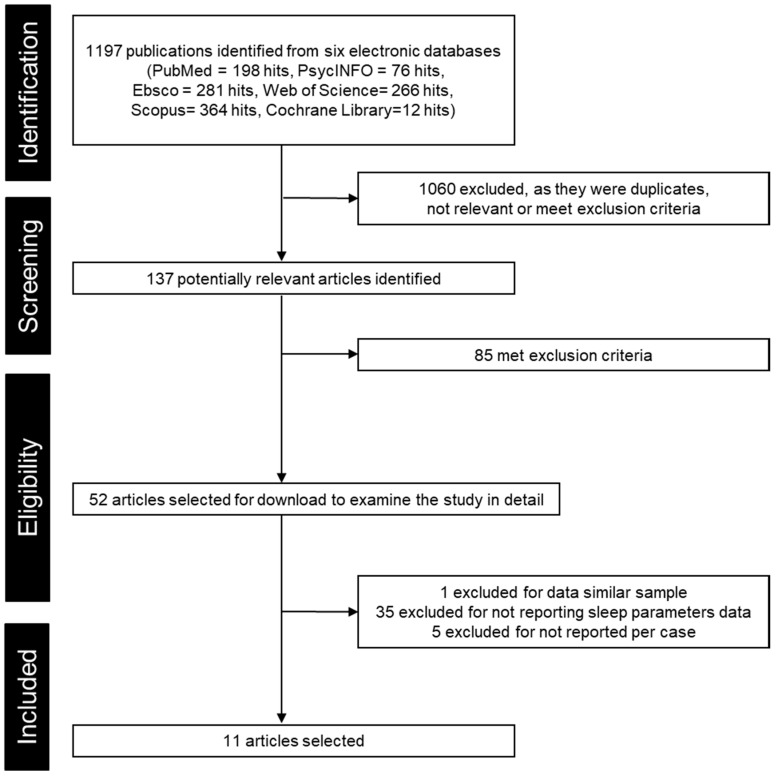
Flowchart of article selection up to the date of 6 February 2022.

In parallel with our serial reviews [18,19] concerning sleep in RTT following the PRISMA 2009 guidelines [20] (PROSPERO: CRD 42020198099), we extracted all RTT case and case series designs; that is, data were selected from the case and case series studies published in English in peer-reviewed journals on RTT with clinical or genetic diagnosis. The PSG data of those RTT cases should be reported numerically or graphically, allowing measurement as numerical data. Studies published up until 6 February 2022 were included (Figure 1 and Supplemental Table S1). RTT cases reported with other central nervous system complications (e.g., neurofibroma) [21] were excluded.

### 2.2. Data Collection and Analysis

First, both authors extracted and checked study information, sample characteristics and PSG parameter definitions reported originally in the papers (Table 1).

Next, when applicable, we generated parameters per TST based on the available data and reported PSG parameters of stages in the sleep macrostructure (i.e., NREM sleep stages 1, 2 and 3 and REM sleep) as the percentage ratio of TST. The definitions of the retrieved PSG parameters across studies are: (1) TIB: time in bed, time from ‘lights off’ in the evening to ‘lights on’ in the next morning; (2) TST: total sleep time, the time from sleep onset to the end of the final sleep epoch minus time awake; (3) SOL: sleep onset latency, time from lights off to sleep onset; (4) SEI: sleep efficiency, the percentage ratio of TST/TIB*100; (5) WASO: wakefulness after sleep onset, the time spent awake between sleep onset and end of sleep; (6) REM(%) stage: rapid eye movement sleep, proportion of REM sleep per TST; (7) NREM(%) stage: non-rapid eye movement sleep was further divided into stage 1 (N1), stage 2 (N2), and stage 3 (N3, also reported as the slow-wave sleep, SWS; i.e., includes stage 4 if reported separately), proportion of each NREM stage per TST; (8) AHI: apnea/hypopnea index per hour of TST, normal value ≤1/h [22]; (9) OAI: obstructive apnea index per hour of TST; (10) CAI: central apnea index per hour of TST; *(*11*)* ODI: oxygen desaturation index per hour of TST; (12) OAHI: obstructive apnea hypopnea index per hour of TST; (13) SpO_2_% mean: mean oxygen saturation (%); (14) SpO_2_% nadir (lowest): minimal oxygen saturation (%).

Lastly, cases were classified based on available information regarding genes, presence of epilepsy and scoliosis and history of adeno(tonsil)ectomy (A*&*T) for stratified analysis. Strata were further compared to sleep data for typically developing individuals from the literature [23] (see in Table 2). We performed a sensitivity analysis by excluding in-home sleep studies and A*&*T surgery cases.

### 2.3. Statistical Analysis

We report here the descriptive results (means ± standard deviations) for the total sample (i.e., combination of all literature cases) and the RTT strata. Kruskal–Wallis ANOVA tests by rank were applied to examine the differences among strata within each stratification. Statistical analysis with standardized mean difference (SMD) calculations were used to measure the mean differences between RTT groups and sleep data reported for typically developing individuals in the literature [23]. To assess the relationship with chronological age at PSG, Spearman’s correlation coefficient test was applied. 

Statistical analyses were performed in IBM SPSS Statistics, version 25.0. Here, *p*-values are shown and significance was to set at *p* < 0.05.

The Study Quality Assessment Tools of the National Institutes of Health (NIH) [24] applicable to several study designs were used for scoring study quality [25,26].

**Table 1:** Demographic information for the studies selected.

Author(Year)	Country	Gender: n	Age Mean ± SD [Range], y	Gene (n)	Classification, Stage(n)	Diagnosis	Sleep Assessment Tool	Sleep Scoring Guideline	Sleep Issue Investigated	Type of Study
Cacciatori et al., 2020 [27]	Italy	M: 1	0.8	*MECP2*		C, G	Home sleep test	AASM Scoring Manual Version 2.2	SDB	Case report
Sarber et al., 2019 [28]	United States	M: 2 F: 11	10.3 ± 4.9, [2.6–17.4]	*MECP2* (11)	Classic	C, G	PSG	AASM 2007–2017	SDB, sleep structure	Case-series
Amaddeo et al., 2019 [13]	France	F:17	9.5 ± 2.8 [6–16]	*MECP2* (11)		G	PSG	AASM 2007	SDB, sleep structure	Case-series
Ohno et al., 2016 [29]	Japan	F: 1	6.6	*MECP2*		G	PSG	?	SDB, sleep structure	Case report
Bassett et al., 2016 [30]	United States	F:14	7.8 ± 4.9, [1.9–17.6]				PSG	?	SDB	Case-series ^3^
Hagebeuk et al., 2012(a) [31]	the Netherlands	F:12	9.5 ± 8.8, [3–33]	*MECP2 (*9)	III (9) and IV(1)	C [6], G	PSG	AASM 2007	SDB	Case-series
Hagebeuk et al., 2012(b) [11]	the Netherlands	F:4	6.5 ± 5.8, [2–15]	*CDKL5*		G	PSG	AASM 2007	SDB, sleep structure	Case-series
d’Orsi et al., 2009 [32]	Italy	F: 1	2	*MECP2*		G	PSG ^1^	AASM	EEG, SDB	Case report
Schluüter et al., 1995 [33]	Germany	F:2	13 ± 5.7, [9, 17]				PSG ^2^	Schlüter 1993	SDB, EEG, sleep structure	Case-series
Aldrich et al., 1990 [34]	United States	F: 4	7.0 ± 3.0, [4–11]		III (4)	C [35]	PSG	Rechtschaffen and Kales 1968	EEG, sleep structure, SDB	Case-series
Nomura et al., 1985 [15]	Japan	F: 5	5.8 ± 4.4, [2–12]				PSG	Segawa?	EEG, sleep structure	Case-series

**AASM:** American Academy of Sleep Medicine; **C:** clinically; ***CDKL5*:** cyclin-dependent kinase-like 5; **ECG:** electrocardiography; **EEG:** electroencephalogram; **F:** female; **G:** genetically; **M:** male; ***MECP2*:** methyl-CpG-binding protein-2; **PSG:** polysomnography; **SD:** standard deviation; **SDB:** sleep-disordered breathing. ^1^ Video polygraphy included EEG (electrodes placed according to the 10–20 International system with bipolar montage), electromyogram from submental muscle, the right deltoid muscle, the right and left flexor and extensor muscles of the hand and both muscles tibialis anterior; EKG; oronasal (monitored with a thermistor), thoracic and abdominal respiration (monitored with a strain gauge); electrooculogram; oxygen saturation, heart rate and pulse and AASM manual for the scoring of sleep and associated events. ^2^ Polygraphic technique included electroencephalogram, electro-oculogram, nasal airflow, thoracic and abdominal breathing movements, electrocardiogram and transcutaneous oxygen saturation. ^3^ Correspondence letter.

**Table 2:** Summary of strata comparisons.

Parameters			Age (year)	TST (min)	SEI (%)	WASO (min)	SOL (min)	N1 (%)	N2 (%)	N3 (%)	REM (%)
**Typically developing individuals**		mean ± SD	9.39 ± 5.50	490.94 ± 26.56	89.53 ± 2.59	32.06 ± 14.27	25.81 ± 5.73	7.15 ± 0.52	39.69 ± 6.56	30.40 ± 4.84	21.32 ± 2.10
		n	209	209	209	209	209	209	209	209	209
**Total RTT group**		mean ± SD	8.38 ± 3.88	417.30 ± 154.18	70.14 ± 21.84	189.21 ± 165.06	23.56 ± 45.62	9.51 ± 10.35	34.51 ± 16.83	41.05 ± 21.19	14.74 ± 9.28
		n	33	27	27	27	8	30	30	31	31
		SMD (p)		**1.29 (0.000)**	**2.53 (0. 000)**	**−2.77 (0.000)**	0.23 (0.53)	**−0.65 (0.001)**	**0.61 (0.002)**	**−1.21 (0.000)**	**1.72 (0.000)**
**Gene**	***MECP2***	mean ± SD	8.79 ± 2.28	368.20 ± 89.19	65.93 ± 20.07	223.97 ± 180.98		1.70 ± 1.71	32.47 ± 16.86	51.93 ± 19.90	13.03 ± 9.58
		n	16	15	15	15		15	15	16	16
		SMD (p)		**3.60 (0.000)**	**4.19 (0.000)**	**−4.04 (0.000)**		**8.22 (0.000)**	**0.95 (0.000)**	**−3.09 (0.000)**	**2.58 (0.000)**
	***CDKL5***	mean ± SD	6.50 ± 5.80	666.23 ± 110.38	70.58 ± 11.69	238.97 ± 98.98	38.80 ± 63.25	25.01 ± 4.28	43.34 ± 14.89	22.77 ± 13.15	8.88 ± 6.58
		n	4	4	4	4	4	4	4	4	4
		SMD (p)		**−5.95 (0.000)**	**6.47 (0.000)**	**−11.22 (0.000)**	**−1.38 (0.01)**	**−24.57 (0.000)**	−0.54 (0.28)	**1.51 (0.003)**	**5.58 (0.000)**
	Missing	mean ± SD	8.46 ± 4.88	384.90 ± 159.35	77.81 ± 28.32	99.15 ± 134.94	8.33 ± 15.39	14.52 ± 9.26	34.10 ± 17.88	31.87 ± 16.92	19.36 ± 8.12
		n	13	8	8	8	4	11	11	11	11
K-W ANOVA test between gene strata				**H (1, N = 19) = 9.00 *p* = 0.003**	H (1, N = 19) = 0.09 *p* = 0.73	H (1, N = 19) = 0.81 *p* = 0.37		**H (1, N = 19) = 9.29 *p* = 0.002**	H (1, N = 19) = 1.21 *p* = 0.27	**H (1, N = 20) =6.51 *p* = 0.01**	H (1, N = 20) = 1.08 *p* = 0.30
**Epilepsy**	**Not have**	mean ± SD	8.23 ± 2.04	405.67 ± 37.23	82.33 ± 8.39	87.38 ± 41.96		1.17 ± 0.29	38.33 ± 14.01	41.33 ± 14.01	18.67 ± 7.23
		n	3	3	3	3		3	3	3	3
		SMD (p)		**3.20 (0.000)**	**2.66 (0.000)**	**−3.74 (0.000)**		**11.50 (0.000)**	0.20 (0.73)	**−2.18 (0.000)**	**1.20 (0.04)**
	**Have**	mean ± SD	8.65 ± 3.66	430.64 ± 153.8	69.67 ± 19.94	200.93 ± 168.11	23.56 ± 45.62	9.58 ± 10.95	35.73 ± 18.57	42.52 ± 24.4	11.73 ± 8.61
		n	22	22	22	22	8	22	22	22	22
		SMD (p)		**1.14 (0.000)**	**3.04 (0.000)**	**−3.20 (0.000)**	0.23 (0.53)	**−0.72 (0.001)**	**0.47 (0.04)**	**−1.39 (0.000)**	**2.92 (0.000)**
	Missing	mean ± SD	7.23 ± 4.60	288 ± 284.26	57.01 ± 55.77	213 ± 275.77		14.23 ± 8.192	26.88 ± 7.32	35.48 ± 8.29	23.81 ± 6.04
		n	16	2	2	2		5	5	6	6
K-W ANOVA test between epilepsy strata				H (1, N = 25) = 0.06 *p* =0.80	H (1, N = 25) = 1.28 *p* = 0.26	H (1, N = 25) = 2.02 *p* = 0.16		H (1, N = 25) = 1.03 *p* = 0.31	H (1, N = 25) = 0.11 *p* = 0.74	H (1, N = 25) = 0.02 *p* = 0.90	H (1, N = 25) = 1.37 *p* = 0.24
**Scoliosis**	**Have**	mean ± SD	9.48 ± 2.76	365.82 ± 102.02	66.24 ± 19.48	212.97 ± 172.35		2.21 ± 3.02	34.29 ± 20.28	50.53 ± 23.33	12.35 ± 9.16
		n	17	17	17	17		17	17	17	17
		SMD (p)		**3.35 (0.000)**	**4.03 (0.000)**	**−3.76 (0.000)**		**5.20 (0.000)**	**0.65 (0.01)**	**−2.58 (0.000)**	**2.82 (0.000)**
	Missing	mean ± SD	7.23 ± 4.60	504.81 ± 191.46	76.78 ± 25.01	148.81 ± 151.71	23.56 ± 45.62	19.06 ± 8.50	34.80 ± 11.67	29.53 ± 10.29	17.64 ± 8.89
		n	16	10	10	10	8	13	13	14	14
**A*&*T history**	**Not have**	mean ± SD	8.67 ± 2.07	364.91 ± 105.17	63.91 ± 23.08	249.69 ± 205.99		131.64 ± 1.63	27.73 ± 15.82	57.27 ± 19.87	12.64 ± 10.77
		n	11	11	11	11		11	11	11	11
		SMD (p)		**3.67 (0.000)**	**4.61 (0.000)**	**−4.70 (0.000)**		**8.92 (0.000)**	**1.65 (0.000)**	**−4.23 (0.000)**	**2.81 (0.000)**
	**Have**	mean ± SD	9.94 ± 2.68	404.40 ± 61.33	73.80 ± 7.92	143.61 ± 46.96		1.50 ± 2.06	39.40 ± 17.98	45.80 ± 20.01	12.80 ± 6.14
		n	5	5	5	5		5	5	5	5
		SMD (p)		**3.13 (0.000)**	**5.64 (0.000)**	**−7.18(0.000)**		**9.59 (0.000)**	0.04 (0.93)	**−2.79 (0.000)**	**3.79 (0.000)**
	Missing	mean ± SD	7.74 ± 4.94	475.56 ± 205.93	74.71 ± 24.70	149.46 ± 143.94	23.56 ± 45.62	18.56 ± 8.38	38.10 ± 16.68	27.56 ± 12.51	16.93 ± 9.00
		n	17	11	11	11	8	14	14	15	15
K-W ANOVA test between A*&*T history strata				H (1, N = 16) = 0.03 p =0.87	H (1, N = 16) = 1.04 p = 0.31	H (1, N = 16) = 1.16 p = 0.28		H (1, N = 16) = 0.06 p = 0.82	H (1, N = 16) = 1.41 p = 0.23	H (1, N = 16) = 0.93 p = 0.34	H (1, N = 16) = 0.03 p = 0.87

Bold are significant results. **A***&***T:** adeno(tonsil)ectomy surgery; ***CDKL5*:** cyclin-dependent kinase-like 5; **K-W** **ANOVA:** Kruskal-Wallis one-way analysis of variance; ***MECP2*:** methyl-CpG-binding protein-2; **n:** number; **N1 (%):** percentage of non-rapid eye movement sleep stage 1 of TST; **N2 (%):** percentage of non-rapid eye movement sleep stage 2 of TST; **N3 (%):** percentage of non-rapid eye movement sleep stage 3 of TST; ***p***: *p*-value; **SEI:** sleep efficiency; **SOL:** sleep onset latency; **SD:** standard deviation; **SMD:** standard mean difference; **TST:** total sleep time; **RTT:** Rett syndrome; **REM (%):** percentage of rapid eye movement sleep of TST; **WASO:** wake after sleep onset.

## 3. Results

### 3.1. Cases Extracted from the Literature

We extracted in total 74 cases from eleven studies, of whom 33 had data for sleep macrostructure analyses and 69 had data for sleep respiratory analyses. The ages ranged from nine months to thirty-three years old (8.7 ± 5.1 years), including three males from two studies (Table 1). No study fully reported RTT cases in accordance with the syndrome guidelines published in 2001 [36].

**Figure 2:**
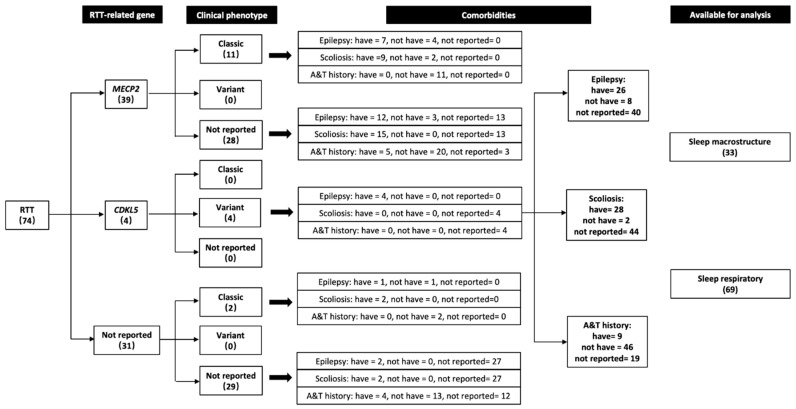
Number of cases in stratification analysis. **A***&***T:** adeno(tonsil)ectomy surgery; ***CDKL5*:** cyclin-dependent kinase-like 5; ***MECP2*:** methyl-CpG-binding protein-2; **RTT:** Rett syndrome.

Regarding the available clinical information (Figure 2), 13 cases were of known classic phenotype and four of variant type, 14 in stage III and two in stage IV of disease progression. Regarding genetic characteristics, two genes were reported with scores of 39 in the *MECP2* stratum and four in the *CDKL5* stratum, and exact genotypes were known in 32 cases. Two kinds of comorbidities were often reported, such that 30 out of 38 cases involved epilepsy; that is, in 17 cases the usage of antiepileptic drugs was reported. Scoliosis was reported in 28 out of 30 cases. Regarding the sleep intervention for breathing difficulties, 9 out of 55 cases were reported to have an A*&*T history.

### 3.2. Standard Sleep Macrostructure Parameters 

The results of the sleep macrostructure (stratified) analysis and SMD comparison with typically developing individuals per *gene, epilepsy, scoliosis* and *A&T history* case are summarized in Table 2. Of note, we could only extract limited cases who had scoliosis, and SOL was inconsistently reported.

#### 3.2.1. TST (min)

Only the average TST of “*CDKL5*” was longer than “*MECP2*”.

Compared to typically developing individuals, TST in all RTT strata was significantly shorter, except for the *CDKL5* stratum, for which it was longer.

#### 3.2.2. SEI (%)

There was no significant difference in the SEIs within any of the RTT strata. 

RTT total group and all strata had lower SEIs than typically developing individuals, particularly in those stratified into “*MECP2*”, “*have epilepsy*” and “*not have A&T history*”.

#### 3.2.3. WASO (min)

WASO showed no differences in the stratified analyses, and was uniformly longer than in typically developing individuals.

#### 3.2.4. SOL (min)

Limited strata data could be extracted from the selected papers, other than for cases with “*CDKL5*” and “*have epilepsy*” (see in Table 2). Comparisons to typically developing individuals showed that SOL in those two available strata combined as the total RTT group was not significantly different. However, separately, cases with *CDKL5* mutations had significantly longer SOL.

#### 3.2.5. N1 (%) Stage

Only the gene strata comparison of cases showed that cases with the mutant *CDKL5* gene had significantly more stage N1 than “*MECP2*”. When compared to typically developing individuals, significantly more stage N1 was found in the total RTT group and “*CDKL5*” stratum (Table 2). Less stage N1 was found in all strata apart from the “*have epilepsy*” stratum.

#### 3.2.6. N2 (%) Stage

The stratified analysis showed no significant difference per classification of *gene*, *epilepsy* and *A&T history*. In comparison with typically developing individuals, stage N2 was significantly lower in RTT total group and the following strata: *MECP2*, *have epilepsy*, *have scoliosis* and *not have A&T history.* The other strata were not different from typically developing children regarding stage N2.

#### 3.2.7. N3 (%) Stage

*MECP2* mutant cases spent more time in stage N3 than “*CDKL5*”, which was the only significant strata comparison. Stage N3 in RTT was always higher than typically developing individuals but lower in the “*CDKL5*” stratum.

#### 3.2.8. REM (%) 

There was no difference identified in REM within RTT strata. REM was uniformly significantly lower than typically developing individuals.

### 3.3. Sleep Respiratory Parameters of Literature Cases

Ten out of the eleven studies reported data related to respiratory events during sleep. Six studies stated the definitions of their parameters, whilst four did this insufficiently or did not do it at all. These ten papers in total reported sleep breathing parameters for sixty-nine cases. In addition, nine cases from three studies were known to have A*&*T history before PSG, while in other cases this information was not provided. Available operationalization data for sleep respiratory indexes in each study are tabulated in Supplemental Table S2.

The descriptive results of total group and strata excluding one case with in-home sleep test and nine with reported A*&*T history are summarized in Table 3. There was no significant difference. The AHI indicates severe sleep-disordered breathing (SDB). Clinically, cases in the strata “*MECP2*”, “*have epilepsy*”, “*have scoliosis*” and “*not have A&T history*” have severe SDB. Although the average saturation fluctuates around the lower normal boundary, the nadir ranges between 83.6% to 90.2%.

### 3.4. Polysomnographic Parameters Correlated to Age

**Figure 3:**
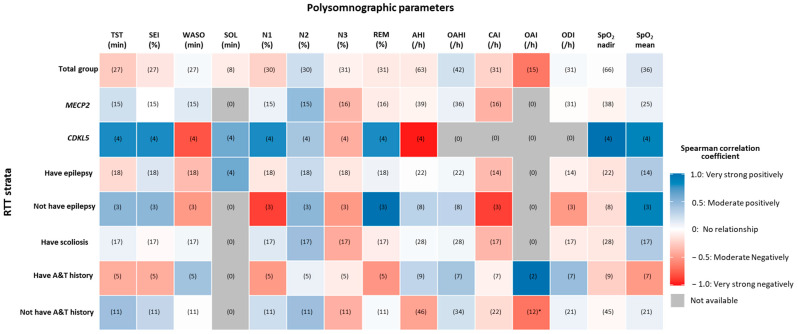
Spearman’s rank correlation of polysomnographic parameters with chronological age in the strata. Numbers of cases are listed in the parenthesis. **AHI:** apnea/hypopnea index per hour of TST, normal value ≤1/h; **A*&*T:** adeno(tonsil)ectomy surgery; **CAI:** central apnea index per hour of TST; ***CDKL5*:** cyclin-dependent kinase-like 5; ***MECP2*:** methyl-CpG-binding protein-2; **N1 (%):** percentage of non-rapid eye movement sleep stage 1 of TST; **N2 (%):** percentage of non-rapid eye movement sleep stage 2 of TST; **N3 (%):** percentage of non-rapid eye movement sleep stage 3 of TST; **SEI:** sleep efficiency; **SOL:** sleep onset latency; **TST:** total sleep time; **OAHI:** obstructive apnea hypopnea index per hour; **OAI:** obstructive apnea hypopnea index per hour of TST; **ODI:** oxygen desaturation index per hour of TST; **REM (%):** percentage of rapid eye movement sleep of TST; **RTT:** Rett syndrome; **SpO_2_% mean:** mean oxygen saturation (%); **SpO_2_% nadir:** minimal oxygen saturation (%), **WASO:** wakefulness after sleep onset. Note: * *p* < 0.05, two-tailed test of significance.

We found no significant association of the polysomnographic parameters with chronological age at PSG in either the RTT total group or any strata (Figure 3). However, in the stratum “*not have A&T history*”, OAI was negatively correlated with age (n = 12, Spearman’s ρ = −0.64, *p* = 0.02).

## 4. Discussion

This is the first study aggregating sleep macrostructure and sleep respiratory data for RTT cases extracted from the literature. Regarding the sleep macrostructure, RTT cases featured shortened TST with increased stage N3 and decreased REM sleep, particularly for the *MECP2* mutant cases. Concerning cases with *CDKL5* mutations, TST was longer and they spent more time in stage N1 but less in stage N3 than those affected by *MECP2* and those typically developing. Severe sleep breathing abnormalities were confirmed. No association with chronological age was found. Therefore, we might conclude that dysregulation of the sleep structure and breathing during sleep occurred since early life in RTT.

### 4.1. Sleep Macrostructure

In our study, in the absence of a reporting done per clinical diagnosis [1,36], we stratified RTT cases published in the literature per RTT-related gene, with the comorbidities of epilepsy and scoliosis and a potential sleep confounder, i.e., A***&***T surgery. We found that in the RTT-related gene strata, the sleep structure and duration showed significant differences despite equivalent SEI and WASO. In particular, N1 was found to be shorter in “*MECP2*” than in “*CDKL5*” cases, while the opposite was true for stage N3. Stages **N2** and REM remained comparable. Although *MECP2* and *CDKL5* are reported to share common molecular pathways [37], it has been suggested that the *CDKL5* subcellular distribution is tightly regulated by the C-terminal tail of *MECP2* [38]. The latter is assumed to result in a milder severity phenotype per Pineda scale [39]. Other strata based on epilepsy, scoliosis and A*&*T history showed no differences. 

#### 4.1.1. Total Sleep Time, Wakefulness after Sleep Onset, Sleep Efficiency

When compared to typically developing individuals some differences were found; that is, the total group of RTT cases showed decreased SEI and impaired sleep continuity. First, the longer WASO and shorter TST (except not for the *CDKL5* stratum) in our results show the poor SEI in RTT. Although in earlier studies SEI was not statistically different from age matched comparison groups [12,14,34,40], the results in more recent studies [13,28] without control groups illustrated lower SEI when compared to a ‘normal’ cut-off. Second, because sleep–wake cycles are partly regulated by the homeostatic system [41], sleep disruptions would subsequently promote sleep, seen as increased homeostatic sleep pressure or sleep drive [42]. Hence, individuals would demonstrate increased daytime sleepiness but also show shortened SOL, longer TST and a greater proportion of deeper sleep during ‘recovery’ nights [43,44]. However, that is not what we found. Although data on SOL were limited, the *CDKL5* stratum had significant longer SOL than typically developing individuals. Overall TST was found to be shorter, except in the *CDKL5* stratum, a finding which could corroborate the increased sleep drive for the *CDKL5* mutant cases. Regarding the stage N3 or deep sleep, increased stage N3 duration was found, except for the *CDKL5* mutant cases. Sleep compensation was suggested in one study involving classic phenotypic RTT girls in stage III or IV compared to a typically developing group [12]. Two earlier studies [14,40] reported indifferent stage N3 results, although these studies were based on comparing RTT with typically developing individuals per age cluster [40] or even with a primary snoring comparison group [14]. Given our *CDKL5* findings within an RTT case sample, the importance of detailed clinical reporting is advised for future sleep studies. Third, sleep state transitions, or the on/off switching of neurons in brainstem regions generating NREM-REM sleep, appear to be controlled by mutually inhibitory neuronal networks [45]. A deficit in one acts almost instantly on the other network, influencing the overall sleep macrostructure.

#### 4.1.2. Stages N1, N2 and N3

Regarding stages N1 and N2, the results were inconsistent in our study, and this variety was mainly seen in the *CDKL5* stratum. More specifically, cases in the *CDKL5* stratum showed a significantly higher stage N1 proportion, which in other RTT strata was lower, than a typically developing sample. Stage N1 is the stage for cortical activities to start slowing down with sleep onset. RTT individuals with *CDKL5* mutations are always characterized by early onset and severe seizures [46], and such highly irregular cortical activities may impact the course of sleep inducement by the brain. Similarly for stage N2, although not significant, the *CDKL5* stratum still showed an increased proportion in this stage compared to a typically developing sample and other RTT strata. Sleep and epilepsy have a complex relationship, particularly during NREM sleep. For instance, slow-wave (N3) sleep differentially activates interictal epileptiform discharges, while during lighter NREM stages (N1, N2) ictal seizure events occur more frequently [47]. Integrated with the significant lower stage N3 proportion in the *CDKL5* stratum than *MECP2* in our results, we may assume that RTT cases of *CDKL5* mutations may result in critical impairment in deep sleep generation, whereas in the RTT cases of *MECP2* mutations this might cause the cessation of deep sleep.

From the pathophysiologic viewpoint, cortical synchrony impairment has been suggested in RTT [48], meaning the changes in sleep macrostructure and sleep state cycling might be attributed to these abnormal cortical activities. In fact, previous studies of electroencephalogram (EEG) spectrum analysis on the one hand reported increased high-amplitude slow waves [49,50,51] during sleep in RTT—potentially prone to epileptic discharges [52], while on the other hand demonstrated the unstable cortical activation related to sleep stage generation and stage maintenance [53,54,55]. As such, NREM parasomnias such as confusional arousals and sleep terrors may occur equally, as reported by caregivers (e.g., night screaming) [8,9,10]. In terms of the potential mechanism, inhibitory processes of neurotransmitter gamma-aminobutyric acid (GABA) receptor [56] are reported to increase the stage N3 proportion during sleep. *Mecp2* mutant mice showed a lack of synapses related to GABAergic neurons [57]; therefore, an increased stage N3 proportion might be reasonably present in RTT girls. However, we require more evidence to clarify the origins regarding increased stage N3 in RTT, given that in *CDKL5* mutant cases stage N3 is reduced.

#### 4.1.3. Rapid Eye Movement Sleep

Lastly, regarding the REM sleep, the finding of attenuated REM was homogeneous across all strata in our aggregated cases. This is a consistent finding in previous polysomnographic studies [12,40], revealing severe REM suppression in RTT. REM sleep is supposed to be engaged in brain development [58,59] and particularly sensorimotor development [60]; that is, newborns spend about half of their sleep time in REM sleep, which continues to decrease to about 20% in adulthood [61]. There is also mounting evidence that REM sleep primes early hippocampal sensorimotor networks towards emerging motor capacities later in life (see the work by Blumberg M. et al.). This lifelong low REM sleep propensity in RTT might not only confirm the damaged brainstem circuits producing REM sleep but likewise may be associated with the severity of sensorimotor deficits seen in RTT. 

With regard to possible mechanisms, during a 24 h in vivo registration in the *Mecp2* mutant mouse model, a glutamate homeostatic dysfunction was suggested [62]. Transmission of endogenous glutamate concentrations was proven to play a role in characterizing the durations of sleep and wakefulness [63]. In human research, fecal glutamate has been shown to be significantly lower in RTT females than unaffected controls [64]. although the specific mechanisms of REM sleep remain unclear [59].

REM sleep reductions are nevertheless often seen in neurodevelopmental disorders such as autism spectrum disorder [65], Down syndrome [66], fragile X syndrome [66], Angelman syndrome [67] and Williams syndrome [68]. Altered sleep-dependent plasticity in timing and space during brain development is proposed as a potential mechanism behind the disordered sleep in these neurodevelopmental disorders [69]. However, and as stated previously, transitions between REM and NREM sleep are under the control of mutually inhibitory neuron populations. Furthermore, *MECP2* is a key factor in regulating synapse formation from early postnatal development [70,71]; hence, such REM sleep suppression in RTT is likely associated with multiple neural molecular pathways. 

In general, the poor sleep continuity in RTT is further supported by the longer WASO duration in our study. This objective finding concurs with their frequent night awakenings reported in surveys [8,9,72]. Intriguingly, upon reviewing polysomnographic studies designed with comparison groups, the RTT group showed significantly enhanced WASO compared to normal peers [12], but not when compared to a primary snoring sample [14]. Studies investigating the respiratory related-arousals are advocated.

### 4.2. Sleep Respiratory Events

Breathing abnormalities are well described in RTT during the wake phase [16,73] but less so during the sleep phase [12]. We demonstrated here that sleep breathing abnormalities are common and severe. As mentioned before, *MECP2* plays an important role in the maturation of nerve cells and in forming synaptic contacts by silencing the transcription of other genes. Furthermore, the MECP2 protein is also variably concentrated in different regions of the brain [74,75,76]. Hence, ventilatory control from the autonomic nervous system could be abnormal at very different levels. Previous respiratory studies [16,17] classified daytime breathing dysrhythmia during the wake state into cardiorespiratory phenotypes, i.e., forceful, feeble and apneustic breathing. These have been linked to the baseline cardiovascular parasympathetic activity governed by brainstem maturity [17]. Studies focusing on sleep breathing in RTT [11,30] indicated that individuals with RTT usually present irregular breathing patterns likely predominated by central apnea, in line with our results. Nonetheless, other studies did not find remarkable sleep breathing abnormalities when compared to age-matched primary snoring [14] or normal [40] groups. Upon stratifying the literature cases by history of A*&*T surgery in this study, we found no significant result, indicating that the sleep breathing abnormalities in RTT might be outweighed by the central control instead of the peripheral airway. However, given the different respiratory indexes analyzed in our study, we may speculate that the disrupted sleep breathing might be determined by dysfunctions on a more global level rather than merely the brainstem. 

### 4.3. Associations with Age

Our results showed that the parameters of sleep macrostructure and sleep breathing had no association with chronological age aside for the OAI. Similarly, cardiorespiratory phenotypes in RTT during the wake phase were reported to be unrelated to age [17]. Although sleep develops dramatically with age [61], as the child grows a morphological aspect at the otolaryngological level might be suspected. Given the non-significant impact of A*&*T surgery, the severe breathing issues seen in RTT likely relate to malfunctioning of the respiratory control system [16]. In contrast, questionnaire studies on RTT report decreasing prevalence rates of sleep problems (i.e., especially night laughing, night screaming and night waking) with age [9]. However, such a decreasing trend has not been reported for sleep-disordered breathing in RTT surveys.

### 4.4. Limitations and Future Scope

Some limitations of this study are noted. The quality of the studies was poor regarding the study population, definitions and sample recruitment, as well as in the interpretation and reporting (Supplemental Figure S1). Sampling bias in this analysis might, therefore, arise from the various study designs and diagnostic criteria applied, leading to weakened validity of our results. In part, our approach was hampered by inconsistency in following the standards in reporting syndromic and sleep variables. As a consequence, and also due to missing data, some sleep macrostructure and sleep respiratory parameters could not be analyzed in more homogeneous strata. Particularly, sleep breathing data were of poor quality because of ambiguous definitions. Moreover, and despite published guidelines, none of the studies applied them when reporting the clinical features of RTT cases [36]. Thus, stratified analysis on such criteria in aggregated cases remains difficult. Lastly, no sleep studies in cases with *FOXG1* mutations were found in the literature, meaning we failed to frame the comprehensive sleep phenotypic landscape regarding RTT-related genes.

Although being a complex syndrome, each case in our study could be fitted on certain RTT-features. However, the stratified analysis can only draw the sleep differences along the strata, which in the absence of stricter adherence to the guidelines will reflect multiple disease characteristics. In addition, and despite combining cases, the sample sizes in several strata were still small (i.e., SOL) or even missing (i.e., not have scoliosis), which may undermine the external validity of the outcomes of our study.

## 5. Conclusions

In this study, we aggregated the literature cases of RTT based on their polysomnographic data for the standard sleep macrostructure and sleep breathing parameters. We firstly revealed the discrepancies in the sleep macrostructure of *MECP2* versus *CDKL5*. Next, we demonstrated severely disturbed breathing during sleep in RTT. In addition, our results suggest that the reported disruptions are consistent from early in life. The shortened TST, enhanced stage N3 and reduced REM sleep may warrant potential targets for future fundamental research in RTT animal models.

## Figures and Tables

**Table 3:** Summary of sleep breathing comparisons.

Parameters			Age	AHI	OAHI	CAI	OAI	ODI	SpO_2_% Nadir	SpO_2_% Mean
**Total RTT group** {* cases excluded}		mean ± SD	8.93 ± 5.15 {8.86 ± 55.37}	12.25 ± 23.89 {11.92 ± 23.67}	6.53 ± 8.67 {6.80 ± 9.30}	6.8 ± 25.09 {8.54 ± 28.40}	1.9 ± 3.91 {2.08 ± 4.81}	18.62 ± 31.9 {19.01 ± 32.48}	84.37 ± 14.42 {85.53 ± 10.85}	96.25 ± 1.89 {96.14 ± 2.02}
		n	69 {59}	63 {53}	42 {32}	31 {24}	15 {13}	31 {23}	66 {56}	36 {29}
**Gene**	***MECP2***	mean ± SD	8.71 ± 5.33	14.77 ± 28.32	6.36 ± 9.06	12.18 ± 34.53	0	19.59 ± 33.82	83.59 ± 11.71	96.07 ± 11.76
		n	39	39	36	16	1	27	38	25
	***CDKL5***	mean ± SD	6.5 ± 5.80	1.48 ± 2.29					90.67 ± 3.21	96.30 ± 0.52
		n	4	4	-	-	-	-	3	3
	Missing	mean ± SD	9.63 ± 4.82	9.49 ± 14.57	7.58 ± 6.33	1.05 ± 1.53	2.04 ± 4.02	12.05 ± 14.20	88.16 ± 9.95	96.77 ± 0.79
		n	26	20	6	15	14	4	25	8
K-W ANOVA test between gene strata				H (1, N = 43) = 3.47 p = 0.06	NA	NA	NA	NA	H (1, N = 42) = 1.22, *p* = 0.27	H (1, N = 28) = 0.13, *p* = 0.91
**Epilepsy**	**Not have**	mean ± SD	9.75 ± 4.22	7.44 ± 6.85	6.29 ± 6.84	0.33 ± 0.58		3.33 ± 3.22	88.50 ± 4.40	97.33 ± 1.16
		n	8	8	8	3	-	3	8	3
	**Have**	mean ± SD	9.87 ± 3.76	17.16 ± 32.78	8.34 ±10.56	13.50 ± 36.89		25.32 ± 40.10	84.45 ±11.94	96.50 ± 2.03
		n	22	22	22	14	-	14	22	14
	Missing	mean ± SD	8.23 ± 5.93	10.15 ± 19.07	3.40 ± 4.52	1.48 ± 1.81	1.90 ± 3.91	15.18 ± 24.98	85.74 ± 11.50	95.89 ± 1.85
		n	39	33	12	14	15	14	36	19
K-W ANOVA test between epilepsy strata				H (1, N = 30) = 0.12, *p* = 0.73	H (1, N = 30) = 0.06, *p* = 0.81	H (1, N = 17) = 0.14, *p* =0.71	NA	H (1, N = 17) = 1.44, *p* = 0.23	H (1, N = 30) = 0.06, *p* = 0.81	H (1, N = 17) = 0.71, *p* = 0.40
**Scoliosis**	**Not have**	mean ± SD	7.0 ± 2.69	4.20 ± 3.96	3.35 ± 3.46				90.20 ± 1.27	
		n	2	2	2	-	-	-	2	-
	**Have**	mean ± SD	10.03 ± 3.84	15.31 ± 29.32	8.10 ± 9.88	11.18 ± 33.65		21.44 ± 37.19	85.19 ± 10.85	96.65 ± 1.90
		n	28	28	28	17	-	17	28	17
	Missing	mean ± SD	8.23 ± 5.93	10.15 ± 19.07	3.40 ± 4.52	1.48 ± 1.81	1.90 ± 3.91	15.18 ± 24.98	85.74 ± 11.50	98.89 ± 1.85
		n	39	33	12	14	15	14	36	19
K-W ANOVA test between scoliosis strata				H (1, N = 30) = 0.21, *p* = 0.65	H (1, N = 30) = 0.17, *p* = 0.68	NA	NA	NA	H (1, N = 30) = 0.69, *p* = 0.41	NA
**A** ***&*T history**	**Not have**	mean ± SD	9.09 ± 5.45	12.46 ± 24.85	6.88 ± 9.43	9.10 ± 29.65	2.25 ± 4.32	18.81 ± 33.89	83.91 ± 11.64	95.96 ± 2.33
		n	46	46	34	22	12	21	45	21
	**Have**	mean ± SD	10.27 ± 2.37	5.80 ± 4.22	5.21 ± 4.56	0.81 ±0.94	0.75 ± 1.06	6.31 ± 7.75	86.89 ±12.79	96.70 ± 1.19
		n	9	9	7	7	2	7	9	7
	Missing	mean ± SD	7.53 ± 5.36	18.30 ± 31.17	4	2.45 ± 3.47	0	45.93 ± 44.28	91.23 ± 2.52	96.61 ± 0.69
		n	14	8	1	2	1	3	12	8
K-W ANOVA test between A*&*T history strata				H (1, N = 55) = 0.05, *p* = 0.83	H (1, N = 55) = 0.10, *p* = 0.76	H (1, N = 29) = 0.03, *p* = 0.87	H (1, N = 14) = 0.08, *p* = 0.78	H (1, N = 28) = 0.86, *p* = 0.35	H (1, N = 54) = 1.46, *p* = 0.23	H (1, N = 28) = 0.44, *p* = 0.51

Bold are significant results. **A*&*T:** adeno(tonsil)ectomy surgery; **AHI:** apnea/hypopnea index per hour of TST, normal value ≤1/h TST; **CAI:** central apnea index per hour of TST; ***CDKL5*:** cyclin-dependent kinase-like 5; **K-W ANOVA:** Kruskal-Wallis one-way analysis of variance; ***MECP2*:** methyl-CpG-binding protein-2; n: number; **OAHI:** obstructive apnea hypopnea index per hour of TST; **OAI:** obstructive apnea index per hour of TST; **ODI:** oxygen desaturation index per hour of TST; ***p***: *p*-value; **RTT:** Rett syndrome; **SD:** standard deviation; **SpO_2_% mean:** mean oxygen saturation (%); **SpO_2_% nadir:** minimal oxygen saturation (%). * Excluded cases assessed by home sleep test and had A*&*T history for sensitive analysis.

## Data Availability

This works is a review paper.

## Supplementary Materials

The following are available online at https://www.mdpi.com/article/10.3390/ijerph19063422/s1: Figure S1: National Institutes of Health (NIH) quality assessment. Table S1: Search items with a cut-off date of 6th February 2022. Table S2: Summarized sleep respiratory event indexes.
