# Supplementary material for: Literature Cases Summarized Based on Their Polysomnographic Findings in Rett Syndrome
Source: Int J Environ Res Public Health. 2022 Mar 14;19(6):3422. doi: 10.3390/ijerph19063422 (PMC8955319; doi:10.3390/ijerph19063422)
Supplement: Supplementary file 1 [file ijerph-19-03422-s001.zip › ijerph-1618073-supplementary.pdf]

International Journal of
Environmental Research and Public Health

MDPI

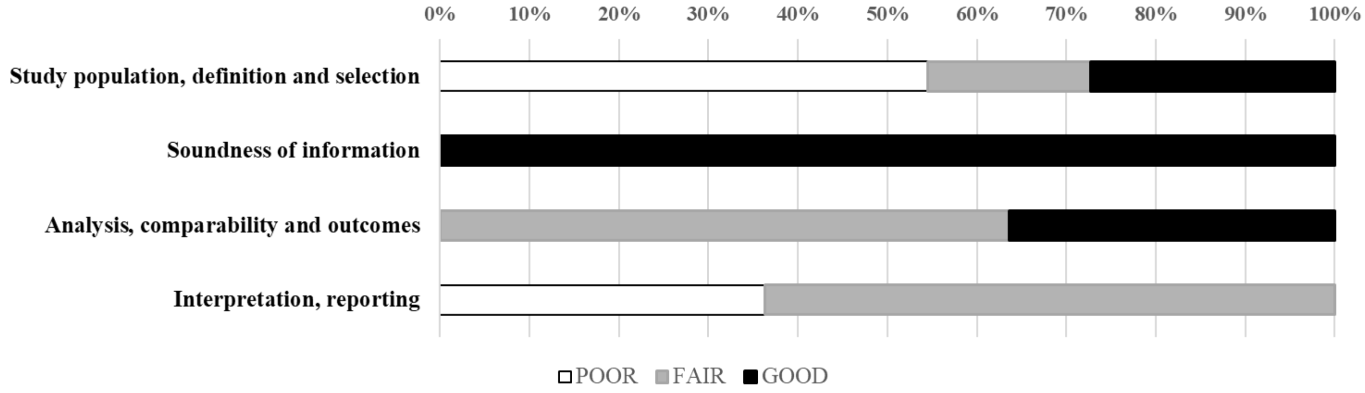

Figure S1. National Institutes of Health (NIH) quality assessment of the 11 studies.

The NIH quality assessment tool is applicable to different study designs and examines each study's internal validity by a set of items. For each study design, we subsequently regrouped items on four domains: "study population, definition and selection", "soundness of information", "analysis, comparability and outcomes" and "interpretation and reporting". A "poor" rating denotes that none of the items within the domain for the respective study design had an affirmative score; a "fair" rating is if less than half of the items were confirmed, and "good" is when more than half of the items were present. We report them as a proportion of the 11 studies examined to reflect the overall quality. Thus, for the 11 studies, a poor rating of more than 50% on the domain "study population, definition and selection" represents mediocre "reporting subject selection method, inclusion/exclusion criteria, sample characteristics etc.".

Table S1. Search items of cut-off date 6th February 2022.

PubMed (total of 198 original citation)	
Search #1	((((((((((((rett syndrome) OR Syndrome, Rett) OR Autism-Dementia-Ataxia-Loss of Purposeful Hand Use Syndrome) OR Autism-Dementia-Ataxia-Loss of Purposeful Hand Use Syndrome) OR (Autism, Dementia, Ataxia, and Loss of Purposeful Hand Use)) OR Rett Disorder) OR Rett's Disorder) OR Rett's Syndrome) OR Retts Syndrome) OR Syndrome, Rett's) OR Cerebroatrophic Hyperammonemia) OR Cerebroatrophic Hyperammonemias) OR Hyperammonemia, Cerebroatrophic) OR Hyperammonemias, Cerebroatrophic (identified 4,193 document citations)
Search #2	Sleep (identified 255,631 document citations)
Search #3	Search #1 AND # 2 ((((((((((((((rett syndrome) OR Syndrome, Rett) OR Autism-Dementia-Ataxia-Loss of Purposeful Hand Use Syndrome) OR Autism-Dementia-Ataxia-Loss of Purposeful Hand Use Syndrome) OR (Autism, Dementia, Ataxia, and Loss of Purposeful Hand Use)) OR Rett Disorder) OR Rett's Disorder) OR Rett's Syndrome) OR Retts Syndrome) OR Syndrome, Rett's) OR Cerebroatrophic Hyperammonemia) OR Cerebroatrophic Hyperammonemias) OR Hyperammonemia, Cerebroatrophic) OR Hyperammonemias, Cerebroatrophic)) AND sleep (identified 198 document citations)
Web of Science (total of 266 original citation)	
Set #1	TOPIC: (rett syndrome) OR TOPIC: (Syndrome, Rett) OR TOPIC: (Autism-Dementia-Ataxia-Loss of Purposeful Hand Use Syndrome) OR TOPIC: (Autism Dementia Ataxia Loss of Purposeful Hand Use Syndrome) OR TOPIC: (Autism, Dementia, Ataxia, and Loss of Purposeful Hand Use) OR TOPIC: (Rett Disorder) OR TOPIC: (Rett's Disorder) OR TOPIC: (Rett's Syndrome) OR TOPIC: (Retts Syndrome) OR TOPIC: (Syndrome, Rett's) OR TOPIC: (Cerebroatrophic Hyperammonemia) OR TOPIC: (Cerebroatrophic Hyperammonemias) OR TOPIC: (Hyperammonemia, Cerebroatrophic) OR TOPIC: (Hyperammonemias, Cerebroatrophic) Indexes=SCI-EXPANDED, SSCI, A&HCI, CPCI-S, CPCI-SSH, BKCI-S, BKCI-SSH, ESCI, CCR-EXPANDED, IC Timespan=All years (identified 6,210 document citations)
Set #2	TOPIC: (sleep) Indexes=SCI-EXPANDED, SSCI, A&HCI, CPCI-S, CPCI-SSH, BKCI-S, BKCI-SSH, ESCI, CCR-EXPANDED, IC Timespan=All years (identified 327,430 document citations)
Set #3	#2 AND #1 Indexes=SCI-EXPANDED, SSCI, A&HCI, CPCI-S, CPCI-SSH, BKCI-S, BKCI-SSH, ESCI, CCR-EXPANDED, IC Timespan=All years (identified 266 document citations)
Scopus (total of 364 original citations)	

Search #1	(TITLE-ABS-KEY (rett AND syndrome) OR TITLE-ABS-KEY (syndrome, AND rett) OR TITLE-ABS-KEY (autism-dementia-ataxia-loss AND of AND purposeful AND hand AND use AND syndrome) OR TITLE-ABS-KEY (autism AND dementia AND ataxia AND loss AND of AND purposeful AND hand AND use AND syndrome) OR TITLE-ABS-KEY (autism, AND dementia, AND ataxia, AND loss AND of AND purposeful AND hand AND use) OR TITLE-ABS-KEY (rett AND disorder) OR TITLE-ABS-KEY (rett's AND disorder) OR TITLE-ABS-KEY (rett's AND syndrome) OR TITLE-ABS-KEY (retts AND syndrome) OR TITLE-ABS-KEY (syndrome, AND rett's) OR TITLE-ABS-KEY (cerebroatrophic AND hyperammonemia) OR TITLE-ABS-KEY (cerebroatrophic AND hyperammonemias) OR TITLE-ABS-KEY (hyperammonemia, AND cerebroatrophic) OR TITLE-ABS-KEY (hyperammonemias, AND cerebroatrophic)) (identified 5,792 document citations)
Search #2	TITLE-ABS-KEY (sleep) (identified 311,893 document citations)
Search #3	((TITLE-ABS-KEY (rett AND syndrome) OR TITLE-ABS-KEY (syndrome, AND rett) OR TITLE-ABS-KEY (autism-dementia-ataxia-loss AND of AND purposeful AND hand AND use AND syndrome) OR TITLE-ABS-KEY (autism AND dementia AND ataxia AND loss AND of AND purposeful AND hand AND use AND syndrome) OR TITLE-ABS-KEY (autism, AND dementia, AND ataxia, AND loss AND of AND purposeful AND hand AND use) OR TITLE-ABS-KEY (rett AND disorder) OR TITLE-ABS-KEY (rett's AND disorder) OR TITLE-ABS-KEY (rett's AND syndrome) OR TITLE-ABS-KEY (retts AND syndrome) OR TITLE-ABS-KEY (syndrome, AND rett's) OR TITLE-ABS-KEY (cerebroatrophic AND hyperammonemia) OR TITLE-ABS-KEY (cerebroatrophic AND hyperammonemias) OR TITLE-ABS-KEY (hyperammonemia, AND cerebroatrophic) OR TITLE-ABS-KEY (hyperammonemias, AND cerebroatrophic))) AND (TITLE-ABS-KEY (sleep)) (identified 364 document citations)
PsycINFO (total of 76 original citations)	
Search	Any Field: rett syndrome AND Any Field: sleep<br>Search Databases: APA PsycInfo, APA PsycArticles, APA PsycBooks, APA PsycExtra (identified 75 document citations)
EBSCO (MEDLINE) (total of 281 original citations)	
Search	Rett syndrome AND sleep<br>Interface - EBSCOhost Research Databases<br>Search Screen - Advanced Search<br>Database - MEDLINE;Academic Search Premier;Business Source Premier;ERIC;GreenFILE;Library, Information Science & Technology Abstracts;Newspaper Source;Regional Business News;Teacher Reference Center;European Views of the Americas: 1493 to 1750;eBook Collection (EBSCOhost);EBSCO eClassics Collection (EBSCOhost);OpenDissertations;The Belt and Road Initiative Reference Source;CINAHL Complete 281 (total of 281 original citations)
Cochrane Library (total of 12 original citations)	

Search	("Rett syndrome"):ti,ab,kw AND (sleep):ti,ab,kw (total of 12 Trials matching)

Table S2. Summarized sleep respiratory event indexes reported.

Study	Index reported	Apnea	Hypopnea	Obstructive apnea	Central apnea	AHI	OAHI	ODI	SpO_2%mean / nadir
Sarber et al., 2019	AHI, OAHI, AI, SpO_2% nadir	A drop in the baseline airflow signal ≥ 90% for the duration dictated by the type of apnea	A decrease in airflow signal of at least 50% (2007–2012) or 30% (2012–2017) for at least two breath cycles, with a corresponding decrease in arterial oxygen saturation of ≥ 3%, an arousal, or both.	The absence of nasal airflow in the presence of continued respiratory effort lasting ≥ 2 respiratory cycles	The absence of both nasal airflow and respiratory effort lasting more than 20 seconds or ≥ 2 breaths associated with a ≥ 3% arterial oxygen desaturation, arousal, or awakening	Number of apneas and hypopneas per hour of sleep time.	Number of obstructive and mixed apneas and hypopneas per hour of sleep.		Pulse oximetry
Hagebeuk et al., 2012(a)	AHI, OAHI, ODI, SpO_2% mean, SpO_2% nadir	An 80% reduction of the amplitude and breathing frequency with a duration of ≥ 10 seconds	A 50% reduction of breathing amplitude for at least 10 seconds	No airflow and no abdominal-thoracic movement in apnea	No airflow despite chest wall and abdominal movements, caused by upper airway obstruction	Amount of apnea per hour, cutoff was set at 1.	Total number of obstructive apneas and hypopneas per hour	Number of >4% decrease from the baseline SpO_2 per hour	Measured using pulse oximetry.
Ohno et al., 2016	AHI, CAI, OAI, MAI, HI	?	?	?	?	?	?	?	?
Hagebeuk et al., 2012(b)	AHI, SpO_2% mean, SpO_2% nadir	A 90% reduction in the amplitude of airflow with a duration of at least two breaths.	A 50% reduction of breathing amplitude for at least two breaths combined with a decrease of 3% in SpO_2	No airflow, despite chest wall and abdominal movements	No airflow and no abdominal– thoracic movement combined with at least 3% desaturation, or an apnea lasting at least 20 seconds.	Total number of apneas and hypopneas per hour of sleep of cutoff at 1	?	?	Continuously measured using pulse oximetry
Amaddeo et al., 2019	AHI, OAHI, AI, ODI, CAI, SpO_2% mean, SpO_2 % nadir (lowest)	?	?	?	?	Number of apneas and hypopneas per hour of TST.		Number of at least 3% oxygen desaturations (DS) per hour of total sleep time.	Pulse oximetry using the Nonin pulse oximeter and the Masimo pulse oximeter.
Aldrich et al., 1990	SpO_2% mean, SpO_2% nadir	?	?	?	?	?	?	?	?
d'Orsi et al., 2009	AHI, ODI, SpO_2% nadir	?	?	?	?	Apnea plus hypopnea per hour of sleep.	?	?	?
Schluüter et al., 1995	SpO_2 nadir%	?	?	?	?	?	?	?	Transcutaneous blood gases.
Bassett et al., 2016	AHI, CAI, OAI, SpO_2 % nadir	?	?	?	Cutoff was set at 1.	AHI>1	?	?	
Cacciatori et al., 2020	AHI, ODI, SpO_2% nadir	?	?	?	?	Total number of respiratory events per hour of total validated time due	?	Number of ≥3% arterial oxygen desaturations/hour of sleep	?

						to mixed or obstructive events.			

AHI: apnea/hypopnea index per hour of TST, normal value ≤1/h; **AI:** arousal index per hour of TST; **CAI:** central apnea index per hour of TST; HI: hypopnea index; **MAI:** mixed apnea index; **OAHI:** obstructive apnea hypopnea index per hour of TST; **OAI:** obstructive apnea index per hour of TST; **ODI:** oxygen desaturation index per hour of TST; **SpO_2% mean:** mean oxygen saturation (%); **SpO_2% nadir:** minimal oxygen saturation (%)
